# Longitudinal genome-wide methylation study of PTSD treatment using prolonged exposure and hydrocortisone

**DOI:** 10.1038/s41398-021-01513-5

**Published:** 2021-07-13

**Authors:** Ruoting Yang, Changxin Xu, Linda M. Bierer, Janine D. Flory, Aarti Gautam, Heather N. Bader, Amy Lehrner, Iouri Makotkine, Frank Desarnaud, Stacy A. Miller, Marti Jett, Rasha Hammamieh, Rachel Yehuda

**Affiliations:** 1 Medical Readiness Systems Biology, Walter Reed Army Institute for Research, Silver Spring, MD USA; 2 Department of Psychiatry, James J. Peters VA Medical Center, Bronx, NY USA; 3 Department of Psychiatry, Icahn School of Medicine at Mount Sinai, Mount Sinai, NY USA

**Keywords:** Predictive markers, Personalized medicine

## Abstract

Epigenetic changes are currently invoked as explanations for both the chronicity and tenacity of post-traumatic stress disorder (PTSD), a heterogeneous condition showing varying, sometimes idiosyncratic responses to treatment. This study evaluated epigenetic markers in the context of a randomized clinical trial of PTSD patients undergoing prolonged-exposure psychotherapy with and without a hydrocortisone augmentation prior to each session. The purpose of the longitudinal epigenome-wide analyses was to identify predictors of recovery (from pretreatment data) or markers associated with symptom change (based on differences between pre- and post-therapy epigenetic changes). The results of these analyses identified the CREB–BDNF signaling pathway, previously linked to startle reaction and fear learning and memory processes, as a convergent marker predicting both symptom change and severity. Several previous-reported resilience markers were also identified (FKBP5, NR3C1, SDK1, and MAD1L1) to associate with PTSD recovery in this study. Especially, the methylation levels of FKBP5 in the gene body region decreased significantly as CAPS score decreased in responders, while no changes occurred in nonresponders. These biomarkers may have future utility in understanding clinical recovery in PTSD and potential applications in predicting treatment effects.

## Introduction

Plasticity of the epigenome is a mechanism through which environmental exposures can result in sustained alterations in gene expression and protein synthesis [1]. Accordingly, epigenetic modifications have been increasingly studied in association with PTSD, a condition that may result from the effects of extreme stress or trauma, often lasting years or decades following exposure, particularly if left untreated [2]. It has been suggested that relatively stable changes in methylation potentially explain both the chronicity and tenacity of PTSD symptoms, as well as the subjective feeling of persons with this condition that they have failed to return to a pretrauma psychological state [3]. Indeed, numerous studies have identified important epigenetic changes in association with the diagnosis of PTSD, in relation to stress-related genes, and increasingly, have used epigenome-wide strategies to identify methylation changes in a variety of discovered genes and CpG sites [4–11].

These findings suggest that epigenetic markers may constitute important biomarkers for PTSD. Interestingly, persons who develop PTSD can recover from this condition, raising the possibility that some epigenetic changes, originally induced by trauma exposure, also change over time and in response to better circumstances or treatment. Cross-sectional studies are limited in their ability to examine the extent to which epigenetic changes, or biomolecules, pathways and networks that have discriminated trauma survivors with and without PTSD, either predict change in association with recovery, or that themselves change in association with symptom severity. The former markers that predict therapeutic outcome have enormous utility for assessment of disability or treatment planning, while the latter may represent important drugable targets.

In prior studies, we have reported on a strategy of measuring biomarkers before and after psychotherapy in order to better understand whether biological measures associated with PTSD change in association with symptom improvement [12]. The direct manipulation of target symptoms with psychotherapy within a relatively short period of time (weeks to months) permits identification of biomarkers associated with relatively rapid symptom change and treatment-associated recovery. In a pilot study, we reported that methylation of the NR3C1 exon 1 F promoter assessed at pretreatment, predicted treatment outcome, but was not significantly altered in responders or nonresponders to prolonged exposure, a cognitive behavioral therapy for PTSD [12]. In contrast, methylation of the FKBP51 exon 1 promoter region did not predict treatment response, but decreased in association with recovery, in that the subset of treatment responders showed higher gene methylation than nonresponders [12]. Endocrine measures were also associated with these epigenetic markers. While preliminary, these results suggested that distinct genes may be associated with both PTSD prognosis and symptoms improvement. Equally important, the findings provided proof of concept that psychotherapy itself constitutes a form of environmental regulation that may alter epigenetic states.

The current study used a similar approach to collect epigenome-wide data from PTSD patients undergoing the same prolonged-exposure psychotherapy in the context of an augmentation design in which subjects were randomized to receiving a hydrocortisone booster or placebo prior to each session. The results of the augmentation strategy did not reveal superior effects of the hydrocortisone (Hcort) augmentation (data reported elsewhere, paper in review); however, the sample yielded a range of responders and nonresponders permitting a similar assessment of predictors and correlates of treatment response. It was hypothesized that some of the previously identified epigenetic markers associated with cross-sectional studies or longitudinal studies of PTSD development during deployment would predict or associate with recovery (resilience). Using computational analyses of the epigenome-wide data, the data detected candidate CpG loci that were further investigated and compared with other epigenome markers reported in prior studies.

## Results

### Behavior of outcome clinical variables

The PTSD symptom severity reduced after prolonged-exposure sessions given either hydrocortisone or placebo. Thirteen and ten participants returned to PTSD negative upon 3-month follow-up for placebo and hydrocortisone groups, respectively (odds ratio 0.83, two-sided Chi-square test, *p* = 0.768). As shown in Table 1, the CAPS score of the individuals with hydrocortisone treatment averagely dropped 36.6 (or 40.5% of CAPS_T1_), which is more than those with placebo (ΔCAPS = 26.7 or 34.4% of CAPS_T1_) (*t* = 1.35, *p* = 0.182). No difference between responders and nonresponders at T3 associates with age (*p* = 0.469), BMI (*p* = 0.606), or early trauma (*p* = 0.890) at the baseline.

**Table 1:** CAPS total scores for all subjects in Pre-treatment (T1) and Follow-up (T3).

	Pre-treatment (T1)	Post-treatment (T2)	3-month Follow-up (T3)
Placebo [mean (sd) *N*]	80.2 (13.3) *N* = 22	50.9 (22.5) *N* = 21 (7 PTSD- /14 PTSD + )	53.5 (28.2) *N* = 22 (10 PTSD- /12 PTSD + )
Hydrocortisone [mean (sd) *N*]	89.0 (13.0) *N* = 20	53.7 (21.0) *N* = 20 (7 PTSD- /13 PTSD + )	53.0 (23.8) *N* = 20 (10 PTSD- /10 PTSD + )
All [mean (sd) *N*]	84.4 (13.7) *N* = 42	52.2 (21.6) *N* = 41 (14 PTSD- /27 PTSD + )	53.2 (25.9) *N* = 42 (20 PTSD-/22 PTSD + )

### Genes responded at post-treatment follow-up (T3) associate with clinical outcomes (T3-responsive genes)

The methylation levels of 2607 probes, including 1641 unique genes in the nonintergenic region (NIGR), significantly associated with clinical outcomes (responders *N* = 20 vs nonresponders *N* = 22, model 1a, *p* < 0.01). Comparing the methylation levels of nonresponders over responders, nonresponders have greater methylation than responders (56% of DMPs were hypermethylated probes) (Fig. S1).

### Genes responded at pretreatment (T1) predict clinical outcomes (T1-predictive genes)

Using the model 1b, 3247 differential methylated probes (DMPs) (1970 unique NIGR) were identified to distinguish responders (*N* = 20) and nonresponders (*N* = 22) (*p* < 0.01). Opposite to the case at T3, nonresponders have less methylation than responders (44% of DMPs were hypermethylated). Furthermore, 300 of the 1823 hypomethylated probes switched to hypermethylation at T3, while less (107) hypermethylated probes switched. (Fig. S1).

### Genes responded at post treatment (T2) associate with clinical outcomes (T2-responsive genes)

The T1-predictive genes have 2933 or 90% remaining in the same methylation direction at T2, while the T3-responsive genes have 82% showing the same direction at T2. However, the DMPs (based on model 1c) that overlap between T1 and T3 are limited (389 and 286, respectively).

### Enriched biological pathways are highly overlapped between T1 and T3

The DMPs at T1 and T3 were related to the 26 and 20 enriched biological pathways identified by Ingenuity Pathway Analysis (*p* < 0.0005, finite z-score) respectively (Table S1, S2). Surprisingly, 16 of the enriched pathways were overlapped. Moreover, the z-scores of T1-enriched pathways were significantly lower than those of T3 pathways (paired *t*-test *t* = −4.27, *p* = 0.0009, Table S3), and seven hypomethylated pathways switched to hypermethylation, which is consistent to the findings of genes.

### Association between methylation changes and PTSD-symptom severity (symptom-tracing genes)

The association between methylation changes and PTSD-symptom severity was calculated by model 2 (magnitude changes) and model 3 (percentage of changes) among 42 participants available at both timepoints. For the average *p*-value of the two models, there were totally 3113 probes whose changes of methylation levels were significantly associated with the changes of symptom severity (*p* < 0.01). The overall positive associations between methylation and symptom changes were significantly more than negative associations (2072 vs 1041). In other words, less methylation was observed in parallel with decreasing PTSD-symptom severity. About 3.7% of the 3113 DMPs associated with severity changes also associated with (model 2, *p* < 0.01) the cell composition, age, or gender. Table S4 listed the top 10 nonintergenic regions significantly correlated with methylation and PTSD-symptom severity changes but not significantly associated with granulocyte, age, and gender.

The list of DMPs associated with severity changes was subjected to pathway-enrichment analysis using Ingenuity Pathway Analysis (IPA). All fourteen significantly enriched pathways (enrichment *p*-value < 0.0005 and finite z-score) were positively associated with severity changes (Table S5).

### The associations between methylation changes and treatments

Although the participants showed an overall decrease for both hydrocortisone and placebo treatment, 110 out of 3113 severity-associated DMPs also showed a significant difference in treatment. The top 10 CpG sites ranked by the average *p*-value of treatment, CAPS changes, and CAPS percentage changes were listed in Table S6, where MAD1L1 has two probes in the top 10 list.

### Putative markers predict treatment outcomes

**Fig. 1:**
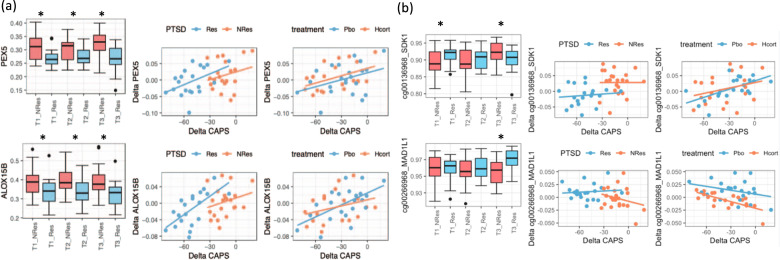
Putative markers predict treatment outcomes. **a** The most significant markers. (Left) Time-course difference between PTSD responders (*N* = 20) and nonresponders (*N* = 22). (Middle) Relationship of methylation between pretreatment (T1) and PTSD-symptom severity at 3-month post treatment (T3) grouped by responders and nonresponders. (Right) Relationship of methylation and PTSD-symptom severity changes grouped by treatment. **b** The reported markers showing the T1 and T2 response or treatment difference (* Statistical significance was set at *p* < 0.05).

We further investigated the putative markers that both differentiate responder status and track PTSD-symptom severity. In this case, the putative markers will be the overlap between T1-predictive, T3-responsive genes, and symptom-tracing genes, which results in two genes, PEX5 and ALOX15B (Fig. 1a). If the significance cutoff of the T1-predictive, T3-responsive genes was relaxed to 0.05, then the number of putative markers improved to 26 (Table 2).

**Table 2:** Putative markers predict treatment outcomes.

Probe	CHR	MAPINFO	Strand	Symbol	Location	CGI
cg17052466	11	2423382	F	TSSC4	TSS200	shore
cg26227186	1	95393138	F	CNN3	TSS1500	shore
cg07152460	11	61583560	F	MIR1908	TSS1500	island
cg11248491	7	6312525	F	CYTH3	TSS1500	island
cg18430895	5	10485607	R		IGR	opensea
cg12514654	7	12537108	F		IGR	opensea
cg08036553	1	64649805	R		IGR	opensea
cg06859084	2	137523042	R		IGR	island
cg21679524	2	98654289	F		IGR	opensea
cg21911343	3	46672436	F		IGR	opensea
cg21265647	13	84480709	F		IGR	opensea
cg18888205	17	8908079	F		IGR	island
cg21670199	15	88801401	R		IGR	shore
cg03155896	6	30879000	F	GTF2H4	Body	shelf
cg11370748	6	153296563	R	FBXO5	Body	opensea
cg00136968	7	4050043	R	SDK1	Body	opensea
cg15028756	12	7343000	R	PEX5	Body	shore
cg21131421	14	68917215	F	RAD51L1	Body	opensea
cg03156546	16	24759640	R	TNRC6A	Body	opensea
cg21239660	17	45006500	R	GOSR2	Body	opensea
cg11780053	19	7268027	R	INSR	Body	shore
cg18298050	22	26829938	F	ASPHD2	Body	island
cg19233757	8	146024629	R	ZNF517	5′UTR	island
cg03732506	17	73088155	F	SLC16A5	5′UTR	shelf
cg15799267	17	7942406	R	ALOX15B	5′UTR	opensea
cg26710347	6	31827226	R	NEU1	3′UTR	shelf

### The pathways predict treatment outcomes

**Fig. 2:**
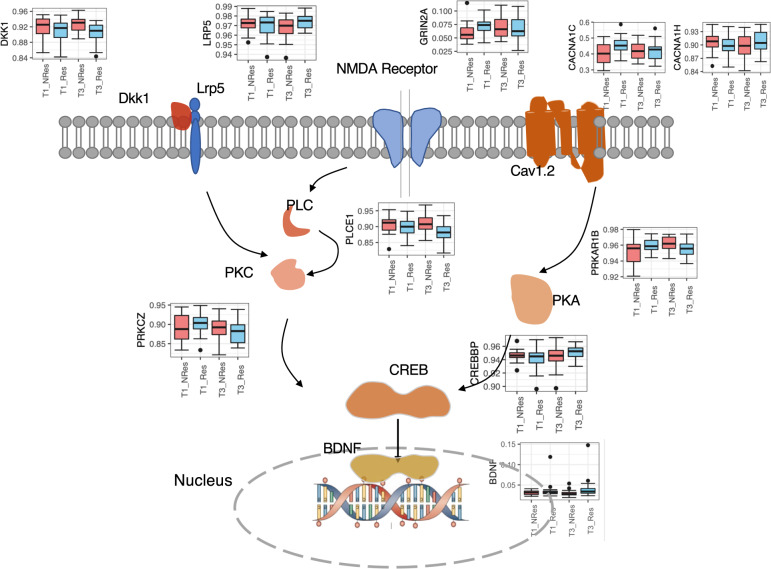
The differentially methylated pathways, identified from the analysis of the DMPs, showed overlap in the Cav1.2-PKA-CREB and DKK1/LRP5-PKC-CREB signaling pathway (nonresponder in red bar, responder in blue bar on the side windows).

Four differentially regulated pathways overlapped among T1-predictive, T3-responsive genes, and symptom-tracing genes (Table S3). In these pathways, six genes were identified among all three categories, and highlighted in the Cav1.2-PKA-CREB and DKK1/LRP5-PKC-CREB signaling pathway (Fig. 2). Six genes had greater methylation in responders than nonresponders at T1, and the methylation level of these genes decreased as the symptom reduced in responders, while the methylation levels of nonresponders increased. It results in a lower methylation of responders than nonresponders at T3. The rest of the three genes have an opposite profile (Fig. 2).

### Observation of NR3C1 and FKBP5 methylation

**Fig. 3:**
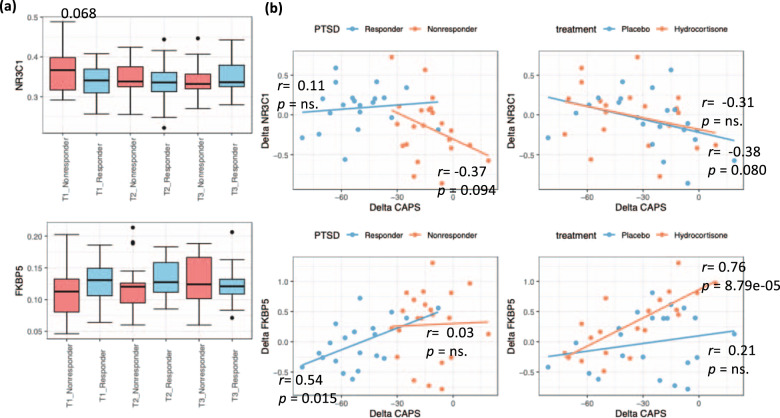
**a** Time-course difference of NR3C1 and FKBP5 methylation between PTSD responders (*N* = 20) and nonresponders (*N* = 22). **b** Relationship of NR3C1 and FKBP5 methylation between pretreatment (T1) and PTSD-symptom severity at three months post treatment (T3) grouped by clinical outcome and treatment.Statistical significance was set at *p* < 0.05.

The pilot study showed the methylation of NR3C1 predicted treatment outcomes, but not the recovery. And NR3C1 decreased in association with recovery but did not predict treatment outcomes. In this study, the methylation levels of NR3C1 had a barely significantly lower methylation in responders than nonresponders at T1 (*p* = 0.068) and was not associated with the recovery. In contrast, the methylation levels of FKBP5 decreased significantly as CAPS score decreased in responders, while no changes occurred in nonresponders. No significant difference between responders and nonresponders was found at T1 or T3. (Fig. 3).

## Discussion

To the best of our knowledge, this is the first study to detect methylation-changes associated changes in PTSD following trauma-focused psychotherapy. A treatment study permits identification of functional diagnostic and prognostic markers of PTSD and resilience because biological correlates of treatment-induced symptom improvement can help distinguish veterans whose symptoms persist from those who recover. There is a paucity of information about the stability of biological networks over time in association with trauma exposure, and even in healthy subjects. These data are critical for interpretation of results from those who recover from PTSD using evidence-based treatment strategies.

In our study, one of the interesting findings is SDK1 (cg00136968) that significantly differentiated responders and nonresponders at both T1 and T3. It increased significantly in nonresponders, and decreased in responders (Fig. 1b). Snijders et al. merged data from US Marine Resiliency Study (MRS) and US Army Study to Assess Risk and Resilience in Servicemembers (Army STARRS), and identified four CpG sites associated with PTSD status changes post deployment, including SPRY4, in SDK1, CTRC, and CDH15 [13]. Among those, the methylation level SDK1 significantly decreased as the PTSD-symptom severity increased. The SDK1 gene was also identified with a CpG–SNP-risk pair in traumatized police officers [14]. This gene has been implicated in schizophrenia [15], autism [16], and MDD [17] in genome-wide association studies.

Large-scale genome-wide association studies have consistently shown that genetic variation in *CACNA1C*, a gene that encodes *calcium voltage-gated channel subunit alpha1C*, increases risk for psychiatric disorders [18], including PTSD [14]. CACNA1C methylation has been implicated in bipolar disorder [19] and suicide attempts patients [20]. The voltage-gated calcium channels have a prominent role in controlling gene expression through coupling membrane depolarization with cAMP-response element-binding protein (CREB) phosphorylation via PKA signaling. CREB can bind to a critical Ca^2+^-response element within brain-derived neurotrophic factor (BDNF) to trigger its transcription. This pathway, and particularly CREB [13,15,] and BDNF [16,17,], were linked to startle reaction and fear-learning and memory processes.

Another interesting finding was in relation to a CpG (cg14284211 chr6: 35570224 near exon 1, cg03591753 chr6: 35659141 near exon) in the body region of the FK506-binding protein 51 gene (FKBP5). This site was found to be significantly decreased as the PTSD symptom decreased in responders, while no change in nonresponders. This is consistent to the finding in the pilot study. Note that the Illumina 450 K chip does not cover on the exact exon regions as we custom-assayed before [12]. Still, FKBP5 acts as a cochaperone that modulates not only glucocorticoid-receptor activity in response to stressors but also a multitude of other cellular processes in both the brain and periphery. The FKBP5 risk allele and child abuse were separately associated with PTSD diagnosis. Specifically, common variants in the FKBP5 gene are associated with higher FKBP5 protein expression, which leads to glucocorticoid receptor resistance and impaired negative feedback in the HPA axis, resulting in a slower return to baseline of stress-induced cortisol levels, which could potentially increase risk for the development of PTSD symptoms [21–23]. The lower methylation in the body region of FKBP5 potentially leads to a lower gene and protein expression, and thus decreases risk of PTSD symptoms.

The treatment difference featured on MAD1L1 gene, encoded mitotic spindle-assembly checkpoint protein MAD1, is a component of the mitotic spindle-assembly checkpoint that prevents the onset of anaphase, until all chromosomes are properly aligned at the metaphase plate. The locus identified on chromosome 7 (rs56226325, MAF = 0.17) near MAD1L1 was previously identified as PTSD-risk locus in GWASs in Million Veteran Program, and also found in bipolar disorder and schizophrenia [24,25,].

The post-treatment T2 seems to be an intermediate stage, since the CAPS changes T3 vs T1 and T2 vs T1 are significantly correlated (*r* = 0.59, *p* = 4.49e-05). The PTSD symptoms and clinical evaluation typically is more stable during the 3-month follow-up. Thus, the discussion in this paper is focused on T3-responsive genes.

The limitations of our study include the following: (1) the sample size of our cohort was relatively small, thus requiring replication in the future using larger samples; (2) the augmentation strategy did not reveal superior effects of the Hcort augmentation; (3) our cohort does not contain civilian PTSD group.

## Conclusion

The epigenome-wide analysis revealed the distinct patterns to associate with the outcome of psychotherapy, which may be explained by a subset of CREB–BDNF pathways. Several previously mentioned resilience markers were also identified. These putative markers may provide useful guidance to predict treatment effect and improve understanding underlying biological mechanisms of PTSD recovery.

## Method

### Cohorts and study design

Participants: The study sample consisted of 88 men and eight women who were previously deployed to Iraq or Afghanistan and who sought treatment for PTSD at the James J Peters VA Medical Center (JJP VAMC). All participants met criteria for deployment-related PTSD according to DSM-IV criteria of greater than 6-month duration, with a minimum score of 60 on the Clinician Administered PTSD Scale for DSM IV (CAPS) [26], and were unmedicated or on a stable psychotropic regimen (i.e., for four weeks or longer). Exclusion criteria were lifetime history of primary psychotic disorder, bipolar disorder or obsessive compulsive disorder, moderate or severe traumatic brain injury, a medical or mental health condition other than PTSD that required immediate clinical attention, substance abuse or dependence within the prior three months, acute suicide risk, psychotropic medication-usage regimen that has not been consistent for four weeks, presence of a medical illness that contraindicates ingestion of a weekly dose of hydrocortisone (e.g., diabetes mellitus), and pregnancy or intent to become pregnant during the study period. The study procedures were approved by the Institutional Review Board (IRB) of the JJP VAMC and Human Research Protection Office (HRPO) from the Department of Defense. All participants provided written informed consent.

### Procedures and measures

Clinical evaluations were performed by independent clinical psychologists at pretreatment (T1), one week post treatment (T2), and after three additional months (T3). The pretreatment evaluation established eligibility and baseline clinical state. T2 and T3 evaluations assessed PTSD symptoms over the past week. The primary outcome measure was PTSD severity as assessed by the CAPS. Current and lifetime psychiatric diagnoses were established by licensed clinical psychologists using the MINI International Neuropsychiatric Interview [27]. A medical evaluation with a licensed nurse practitioner or MD included an assessment of medication usage and medical status and, when indicated, chart review. Moderate or severe TBI was determined by self-reported loss of consciousness following head injury or blast exposure lasting 30 min or longer or altered consciousness lasting greater than 24 s. The presence of likely history of mild traumatic brain injury (mTBI) with clinical indicators of current post-concussive syndrome was established by the clinician-administered VA TBI Screen [28] and a review of the medical record (including any assessment in the JJP VAMC Polytrauma Clinic). Participants also completed clinically relevant self-rated measures to assess potential pretreatment moderators and secondary mood and functional outcomes. The patients who were initially diagnosed as PTSD and switched to non-PTSD after three months are defined as responders, while those who remained PTSD status are called nonresponders. The clinical and demographic characteristics of the subjects are listed in Table 3.

**Table 3:** Clinical and demographic characteristics of the subjects.

	Hydrocortisone (*N* = 25)	Placebo (*N* = 26)	*P*-value
Age, mean ± SD	36.0 ± 8.7	34.8 ± 8.0	0.602
Female (%)	3 (12%)	2 (8%)	0.779
Hispanic, *N* (%)	11 (44.0%)	10 (38.5%)	0.668
BMI, mean ± SD	30.6 ± 5.9	30.9 ± 4.5	0.830
Childhood Trauma^a^	7.8 ± 2.5	8.6 ± 4.0	0.403

PTSD post-traumatic stress disorder. The *p*-values were computed from *t*-test (for Age, BMI, Childhood Trauma) and Fisher’s exact test (for Sex, Race) comparing hydrocortisone to placebo treatment.

^a^Based on Childhood Trauma Questionnaire (CTQ) total score.

Blood was obtained three times at three-month intervals from combat veterans with PTSD at the Icahn School of Medicine at Mount Sinai. The subjects were recruited from the James J Peters Veterans Affairs Medical Centers in the Bronx, New York. All participants received psychotherapy, and half were randomized to receive augmentation with Hcort, and half with placebo. Subjects were diagnosed using the DSM-5 diagnosis, SCID interview, and the Clinician Administered PTSD Scale (CAPS) and other psychological instruments. The enrollment excluded the subjects with the presence of diabetes mellitus or any current unstable medical illness or condition that represents a contraindication to taking glucocorticoids.

### DNA methylation

Blood was drawn by venipuncture in the morning after a night of fasting. Blood draws were collected in PAXgene™ DNA tubes (Preanalytix) and frozen at −80 °C until the DNA extraction. Genomic DNA was extracted from peripheral blood using the PAXgene Blood DNA Kit (Qiagen, Germantown, MD, USA) according to the manufacturer’s instructions followed by quality check using a Tapestation (Agilent). Genomic DNA (500 ng) was treated with sodium bisulfite using the Zymo EZ96 DNA Methylation Kit (Zymo Research, Orange, CA, USA), and genome-wide DNA methylation patterns were profiled using the Infinium HumanMethylation450 BeadChip array (Illumina, Inc., San Diego, CA, USA). BeadChips were washed, single-base extension-labeled, and stained with multiple layers of fluorescence followed by scanning using the Illumina iScan system (Illumina Inc, CA). The samples were randomized and processed in three plates, the samples from the same individual were arranged in one plate. IDAT files containing the raw-intensity signals were generated using Illumina’s iControl software. The methylation level is called a “β value” and for each CpG site, it corresponds to the ratio between the fluorescence signal of the methylated allele (C) and the sum of the fluorescent signals of the methylated (C) and unmethylated (T) alleles. “*β* value” was transferred to “M value” by log_2_(*β*/(1−*β*)) to ensure normal distribution in regression models.

### Quality control and normalization

Genome-wide DNA methylation patterns were profiled using the Infinium HumanMethylation 450 BeadChip (450 K) Kit (Illumina, Inc., San Diego CA, USA). All samples passed the log median-intensity quality control of methylated and unmethylated channels assessed using the R minfi package v1.30.0 [29]. Prior to normalization, probes with low detection (average detection *p*-value >0.01), located on the X and Y chromosomes, that mapped to multiple locations, and/or that colocated with a SNP, were removed using the ChAMP R package v2.14.0 [30], and resulted in 433,378 DNA methylation probes that remained for subsequent analyses. The Quantile plus Beta-Mixture Quantile Normalization (QN.BMIQ) approach was applied to normalize the methylation profiles [31]. The principal component analysis showed no large batch effect between chips. A standard-deviation filter was preapplied to remove the nonchanging probes (sd < 0.01 across all samples), and resulted in 286,100 probes.

### Data analysis

Statistical analyses were performed using R v 3.6.0. Descriptive statistics were calculated for sociodemographic characteristics, clinical assessments, and biological markers where continuous variables were summarized with means ± standard deviations (SD) and independent *t*-test and categorical variables were summarized with frequencies and *χ*^2^ tests. Independent t-tests were conducted to evaluate group differences between PTSD and non-PTSD groups, and a paired *t*-test to compare pre- and post-treatment methylation levels.

### Statistical models

The genes that differentiate responders and nonresponders at post-treatment follow-up (T3) were modeled by a linear-regression model adjusted for age, granulocyte percentage at T3, treatment types, and gender.

Model 1a: Methylation_T3_ ~ Responder + Age + Granulocyte_T3 + Treatment + Gender

Similarly, the genes that predict responders and nonresponders at pretreatment (T1) were modeled by a linear-regression model adjusted for age, granulocyte percentage at T1, treatment types, and gender.

Model 1b: Methylation_T1_ ~ Responder + Age + Granulocyte_T1 + Treatment + Gender

Similarly, the genes that predict responders and nonresponders at post treatment (T2) were modeled by a linear-regression model adjusted for age, granulocyte percentage at T2, treatment types, and gender.

Model 1c: Methylation_T2_ ~ Responder + Age + Granulocyte_T2 + Treatment + Gender

The association between methylation changes (T3–T1) and PTSD-symptom changes was modeled by a linear model adjusted for age, average granulocyte percentage of T1 and T3, treatment types, and gender.

Model 2: △Methylation ~ △CAPS + Age + Granulocyte + Treatment + Gender

To separate the effect of baseline methylation and baseline CAPS levels, the following models were used.

Model 3: (the percentage change of CAPS as a covariate): △Methylation ~ △CAPS /CAPS_T1_ + Age + Granulocyte_T1_ + Treatment + Gender

### Pathway analysis

All the data analysis was conducted under R version 3.6.0. The association between PTSD change and methylation was conducted using Model 1 and statistical significance was defined by *p*-value < 0.01. Ingenuity Pathway Analysis (IPA) (v 01–08, Qiagen, Redwood City, http://www.ingenuity.com) was used to determine functional pathway enrichment, which was defined by a *p*-value < 0.005 and infinite *z*-score.

## Disclaimers

Material has been reviewed by the Walter Reed Army Institute of Research. There is no objection to its presentation and/or publication. The opinions or assertions contained herein are the private views of the author, and are not to be construed as official, or as reflecting true views of the Department of the Army or the Department of Defense. The investigators have adhered to the policies for protection of human subjects as prescribed in AR 70–25.

## Supplementary information

Supplementary Tables

Supplementary Figures

## Data Availability

All datasets for selected cohorts are available with permission through the SysBioCube, at https://sysbiocube-abcc.ncifcrf.gov.
