# Supplementary figures and images for: Longitudinal genome-wide methylation study of PTSD treatment using prolonged exposure and hydrocortisone
Source: Transl Psychiatry. 2021 Jul 13;11:398. doi: 10.1038/s41398-021-01513-5 (PMC8289875; doi:10.1038/s41398-021-01513-5)

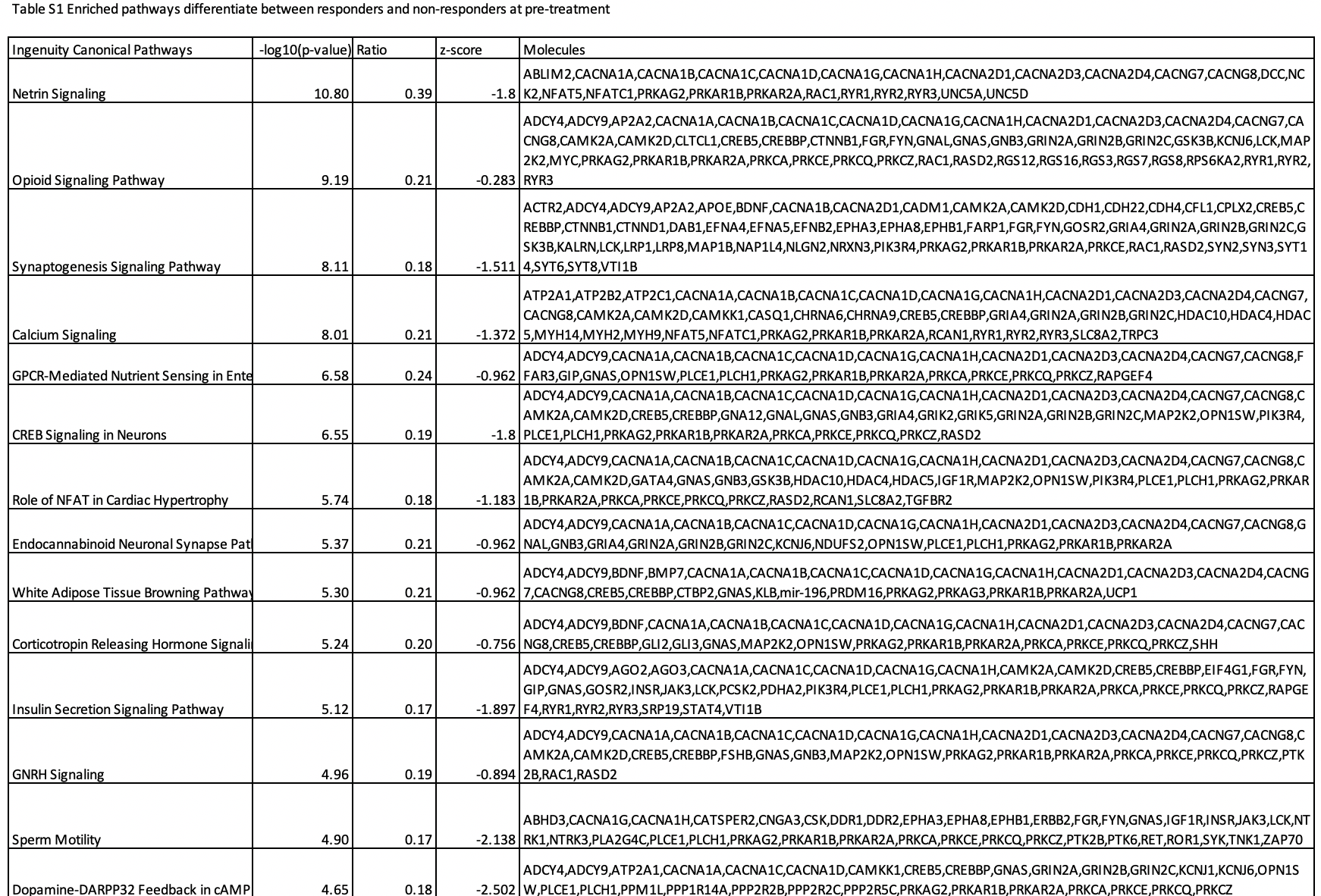


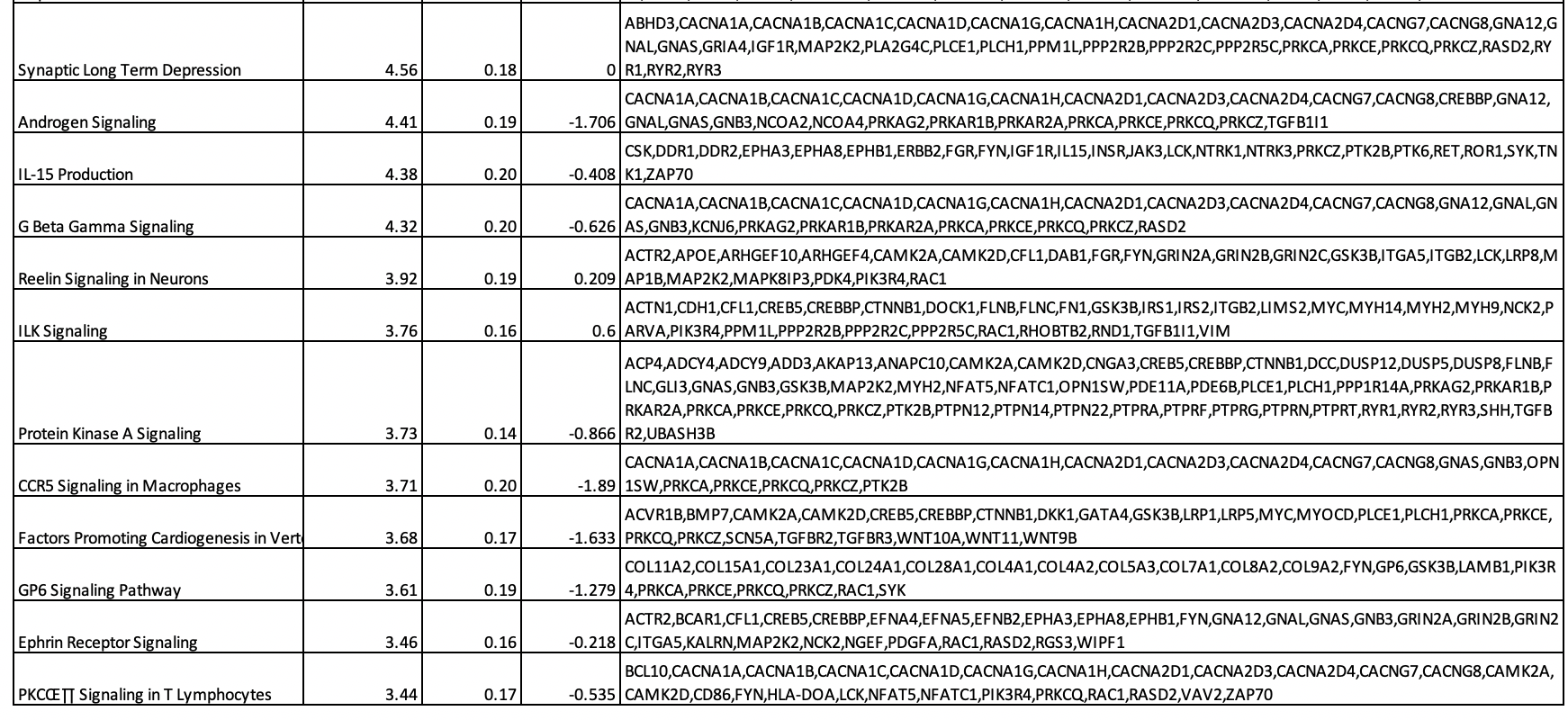


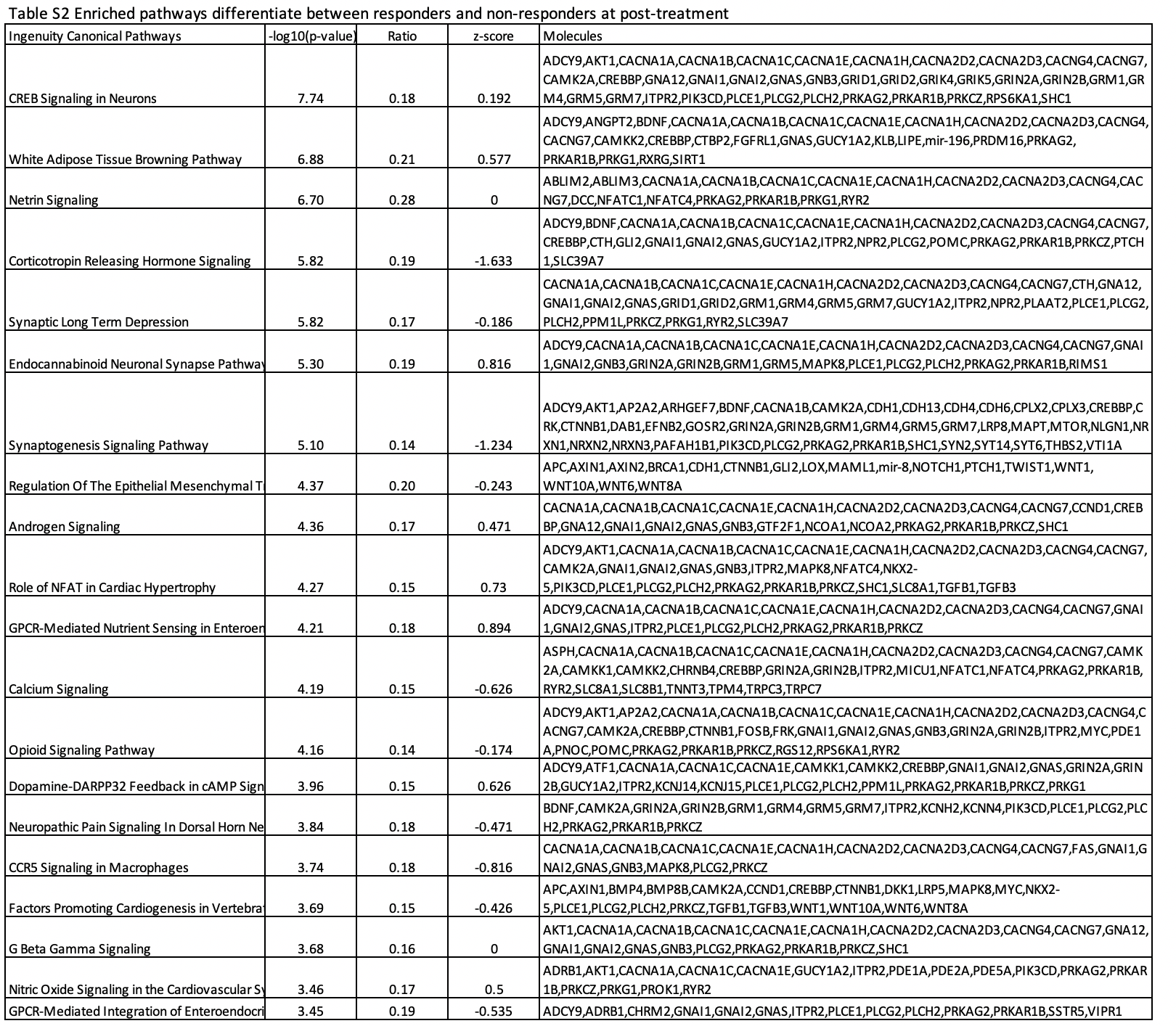


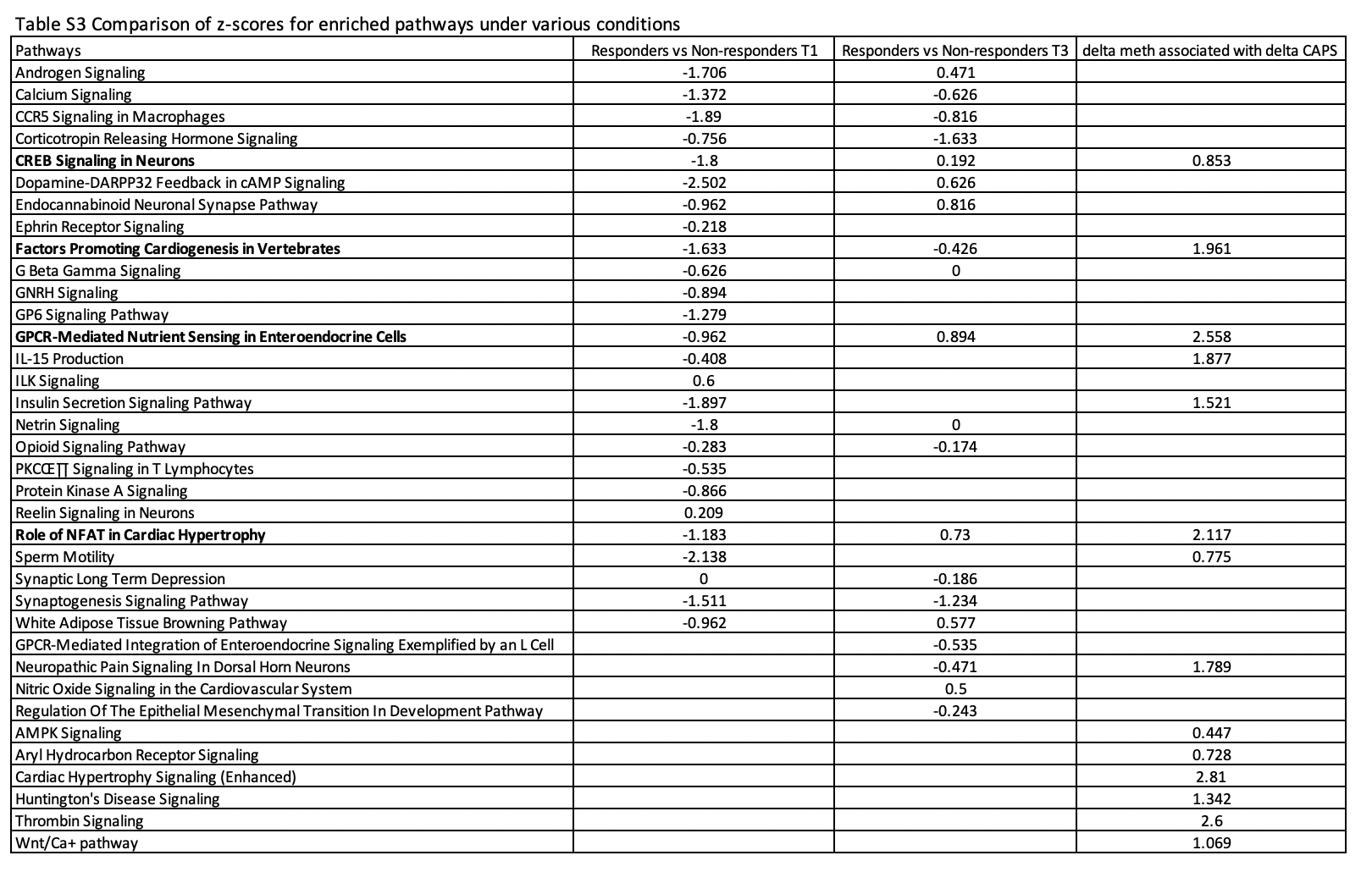


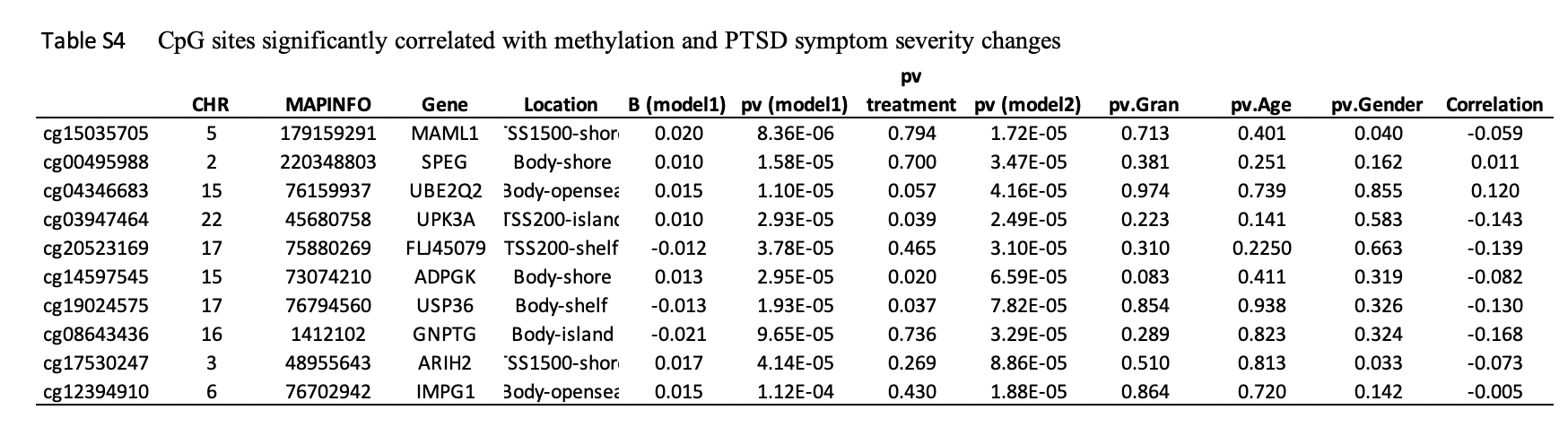


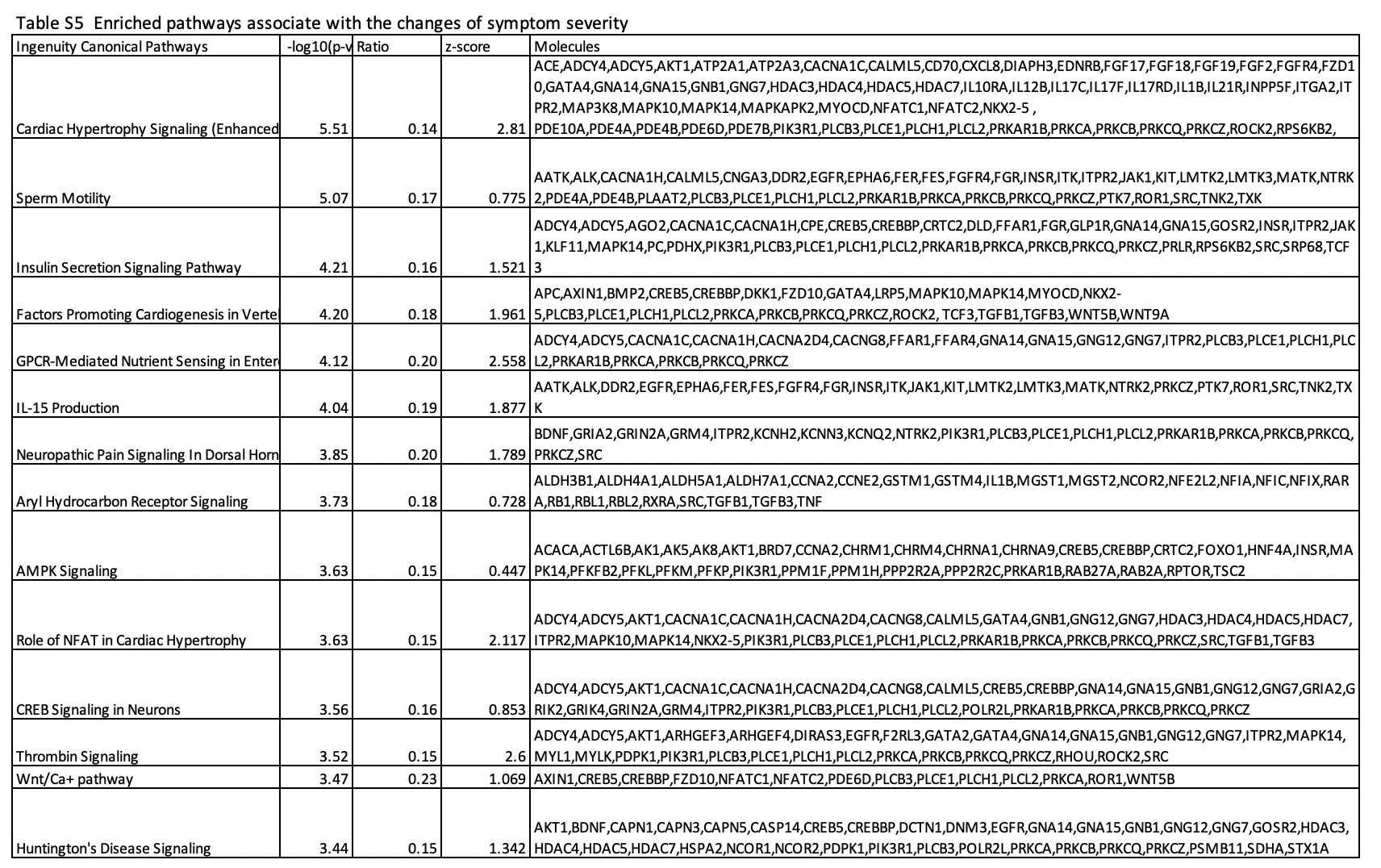


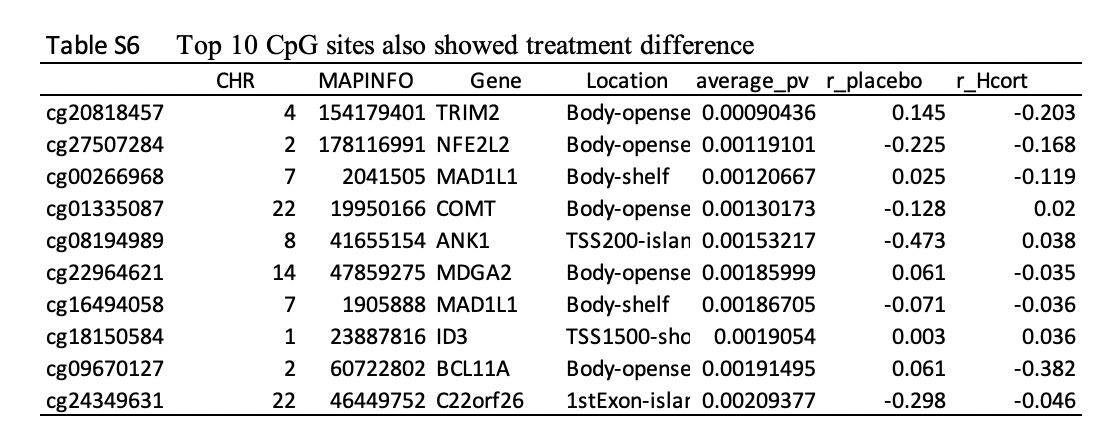

Supplement: Supplementary file 1 — Supplementary Tables [file 41398_2021_1513_MOESM1_ESM.docx]
