# Supplementary material for: Longitudinal genome-wide methylation study of PTSD treatment using prolonged exposure and hydrocortisone
Source: Transl Psychiatry. 2021 Jul 13;11:398. doi: 10.1038/s41398-021-01513-5 (PMC8289875; doi:10.1038/s41398-021-01513-5)
Supplement: Supplementary file 2 — Supplementary Figures [file 41398_2021_1513_MOESM2_ESM.pptx]

## Slide 1
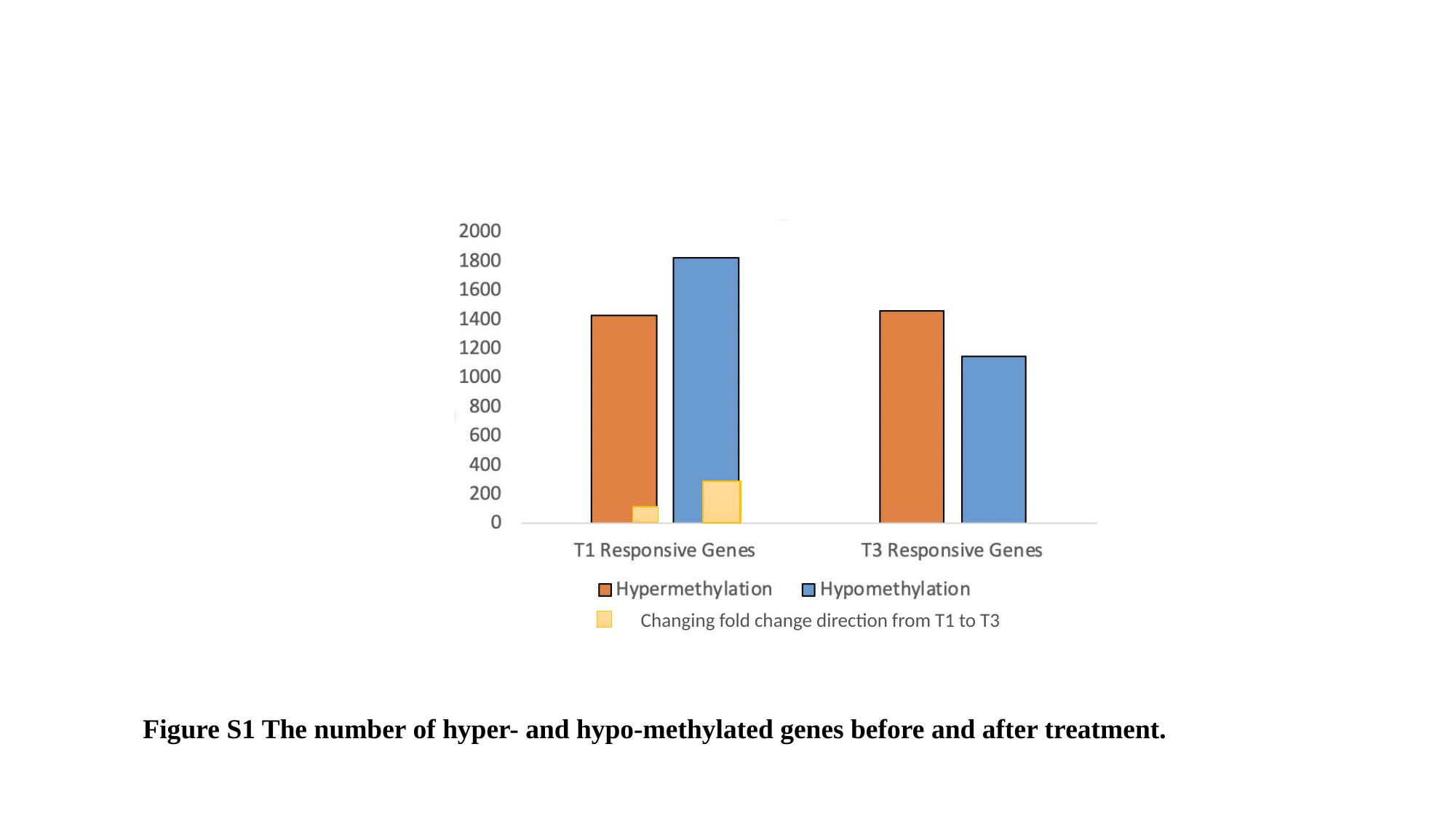

Changing fold change direction from T1 to T3
Figure S1 The number of hyper- and hypo-methylated genes before and after treatment.
